# The Outcomes of Balanced Orbital Decompression for Dysthyroid Optic Neuropathy: Focusing on Choroiretinal Folds with and without Optic Disc Edema

**DOI:** 10.1155/2023/9503821

**Published:** 2023-02-21

**Authors:** Peng Zeng, Xiao-wen Deng, Peng Tian, Yuan-yu Peng, Ming-tong Xu, Shi-you Zhou, Mei Wang

**Affiliations:** ^1^Department of Ophthalmology, Sun Yat-sen Memorial Hospital, Sun Yat-sen University, Guangzhou 510120, China; ^2^Department of Otolaryngology, Sun Yat-sen Memorial Hospital, Sun Yat-sen University, Guangzhou 510120, China; ^3^Department of endocrinology, Sun Yat-sen Memorial Hospital, Sun Yat-sen University, Guangzhou 510120, China; ^4^State Key Laboratory of Ophthalmology, Zhongshan Ophthalmic Center, Sun Yat-sen University, Guangzhou 510060, China

## Abstract

**Purpose:**

To assess the outcomes of balanced orbital decompression for chorioretinal folds (CRFs) with and without optic disc edema (ODE) in dysthyroid optic neuropathy (DON).

**Method:**

A retrospective, interventional study was performed at Sun Yat-sen Memorial Hospital from April 2018 to November 2021. We collected the medical records of 13 patients (24 eyes) with DON and CRFs. Then, we divided them into the ODE group (15 eyes, 62.5%) and the non-ODE group (NODE group, 9 eyes, 37.5%). The valid ophthalmic examination parameters of 8 eyes in each group after balanced orbital decompression were compared at the 6-month follow-up.

**Results:**

The mean best corrected visual acuity (BCVA, 0.29 ± 0.27) and visual field-mean deviation (VF-MD, −6.55 ± 3.71 dB) in the ODE group were significantly worse than those in the NODE group (0.06 ± 0.15 and −3.49 ± 1.56 dB; all *p* < 0.01). Six months after orbital decompression, all parameters were found to have significantly improved in both groups, including BCVA and VF-MD (all *p* < 0.05). Moreover, the improvement amplitude of BCVA (*p* = 0.020) in the ODE group was significantly greater than that in the NODE group. There was no difference in BCVA between the ODE group (0.13 ± 0.19) and the NODE group (0.10 ± 0.13). The disc edema of all eyes (8/8 eyes, 100%) in the ODE group was completely mitigated after orbital decompression. The CRF resolution of 2 eyes (2/8 eyes, 25%) in the ODE group and no eyes in the NODE group was mitigated.

**Conclusions:**

Balanced orbital decompression can significantly improve visual functions and eliminate optic disc edema in DON patients, whether CRF relieves or not.

## 1. Introduction

Dysthyroid optic neuropathy (DON), a serious form of thyroid-associated ophthalmopathy (TAO) that causes optic nerve dysfunction, may result in permanent vision loss; it occurs in 5–8% of all cases of TAO [1–4].

Chorioretinal folds (CRFs), the undulations of the choroid, Bruch's membrane, retinal pigment epithelium, and the overlying neurosensory retina are rarely reported in mild to moderate TAO patients, but they are often found in DON eyes with or without ODE [5–9]. Optic disc edema (ODE), a specific sign of DON, was detected in 56% of DON eyes and was attributed to altered translaminar pressure gradients due to orbital hypertension [10, 11]. These studies highlight that CRFs with ODE may be associated with more visually threatening situations in DON patients than in those with CRFs only. Moreover, Tran et al. confirmed that CRFs persisted after TAO treatment (including orbital decompression) for as long as 2 years in 70% of their eyes [5]. Thus, all of the above findings suggest that the therapeutic effect of orbital decompression in CRFs patients may not be curtly assessed by the presence or absence of CRFs.

To validate this hypothesis, the objectives of this study were to evaluate the clinical features of CRFs with and without ODE in DON patients and report their outcomes after orbital decompression.

## 2. Methods

### 2.1. Subjects

This retrospective, interventional study was conducted at Sun Yat-sen Memorial Hospital, Sun Yat-sen University, from April 2018 to November 2021. This study was conducted in accordance with the tenets of the Declaration of Helsinki, and the protocol was approved by the Sun Yat-sen University Sun Yat-sen Memorial Hospital Ethical Committee in China.

CRFs with ODE (ODE group) and without ODE (NODE group) of DON patients were diagnosed in the Department of Endocrinology and Ophthalmology based on the European Group on Graves' Orbitopathy criteria [12]. The diagnosis of DON was based on decreased visual acuity, a relatively afferent pupillary defect when unilaterally affected, a visual field defect, abnormal pattern visual evoked potential test, and evidence of orbital apex crowding on orbital computed tomography and/or magnetic resonance imaging (MRI) [3, 10]. The activity of TAO is measured by the clinical activity score (CAS), which includes (1) spontaneous retrobulbar pain, (2) pain on attempted upward or downward gaze, (3) redness of the eyelids, (4) redness of the conjunctiva, (5) swelling of the caruncle or plica, (6) swelling of the eyelids, and (7) swelling of the conjunctiva (chemosis). A CAS ≥ 3/7 is indicative of positive activity.

### 2.2. Data Collection

The collected data included age, sex, signs, symptoms, duration of TAO, clinical activity and severity classification of TAO, history and duration of thyroid disease, history of smoking, and the presence of other systemic and eye diseases. Ocular examinations included the best-corrected visual acuity, slit-lamp examination of the anterior segment, and fundus examination with a +90 lens. Proptosis was measured with a Hertel exophthalmometer, axial length was measured with an IOL master, and intraocular pressure (IOP) measurements were obtained in the primary position. Standard automated visual field and pattern visual evoked potentials tests were performed using the Humphrey automated visual field analyzer (program 30-2, Humphrey Field Analyzer II 750; Carl Zeiss Meditec, Inc., Dublin, CA, USA) and the pattern visual evoked potential (ESPION; Diagnosys LLC, Inc., Cambridge, UK), respectively. All patients were examined under a single optical coherence tomography angiography (OCT-A) system (AngioVue; Tovue, Inc., Fremont, CA, USA), which was able to visualize the retinal and choroidal structure at different layers. Some patients with optic nerve edema were examined by fundus fluorescein angiography (FFA, CSLO, Suzhou MicroClear Medical Instruments Co., Ltd., China). The number and proportion of enlarged extraocular muscles (EOMs) per eye and the distance from the optic nerve to the optic strut (optic nerve length) were measured directly on MRI (Phillip, 3.0 T) at each site. The detailed measurement methods were conducted as described.

### 2.3. Treatment before Surgery

Before receiving treatment, a general physical examination was conducted by an endocrinologist (Ming-tong Xu), including routine blood and urine assays, blood biochemistry, blood clotting index tests, liver and kidney function tests, electrocardiographic examination, chest computed tomography (CT), and thyroid function examinations. The therapeutic approaches were based on the recommendations of EUGOGO [12, 13]. Once a diagnosis of DON was made, the high doses of intravenous corticosteroids (500-1000 mg of methylprednisolone) were administered for 3 consecutive days or on alternate days during the 1st week, and urgent orbital decompression was performed if the response was absent or poor within 2 weeks. Recent-onset CRFs should undergo orbital decompression as soon as possible.

### 2.4. Surgical Techniques

Balanced medial plus lateral wall orbital decompression was performed by two of the authors (Mei Wang and Peng Tian). Image-guidance (Fusion Navigation, Medtronic Inc., Jacksonville, FL) was applied to all eyes. Lateral wall orbital decompression was performed much the same as previously reported by an ophthalmologist (Mei Wang) using an eyelid crease incision [14]. The greater wing of the sphenoid bone was removed, and additional removal of the anterior department of the inferior orbital fissure was performed in most patients. Medial wall orbital decompression was performed much the same as previously described by an otolaryngologist (Peng Tian) using a transnasal endoscopic approach [15]. The procedures were as follows: (1) incise and excise the uncinate process. (2) The ethmoid sinus was fully opened, and the lamina papyracea was exposed synchronously. (3) The apertures of the sphenoid sinus and maxillary sinus were adequately opened to prevent inflammation of the sinus cavity. (4) Excision of the lamina papyracea was conducted as much as possible. (5) The periosteum and orbital fascia were cut open to bring about a bulge of orbital fat.

### 2.5. Pre- and Postoperative Evaluation

Valid data of 8 eyes in the ODE and NODE groups after orbital decompression were collected to investigate the outcomes at 6-months follow-up. Before surgery and at 1, 3, and 6 months postoperatively, the patients underwent a complete ophthalmologic examination, including the BCVA, proptosis, IOP, visual field-mean deviation (VF-MD), VF-pattern standard deviation (VF-PSD), CAS, retinal nerve fiber layer (RNFL), and ganglion cell complex layer (GCCL).

### 2.6. Statistical Analysis

Statistical analyses were performed using SPSS (Statistical Package for Social Sciences; SPSS Inc. IBM, Armonk, NY) version 26.0. Continuous variables a represented as the mean ± SD. Categorical variables are expressed in frequency. We take each eye as an independent sample. Considering the data including two eyes of some patients, we used the generalized estimating equation (GEE) method to control the correlation between eyes in regression analysis. For the comparison of classified data, “Binary Logistics” was chosen as the model. For the comparison of grade data, “Ordinal Logistic” was chosen as model. For the comparison of continuous variables, “Linear Regression” is selected as the model. Differences were considered statistically significant at *p* ≤ 0.05.

## 3. Result

### 3.1. Demographic and Clinical Data of DON with CRF Patients

**Figure 1:**
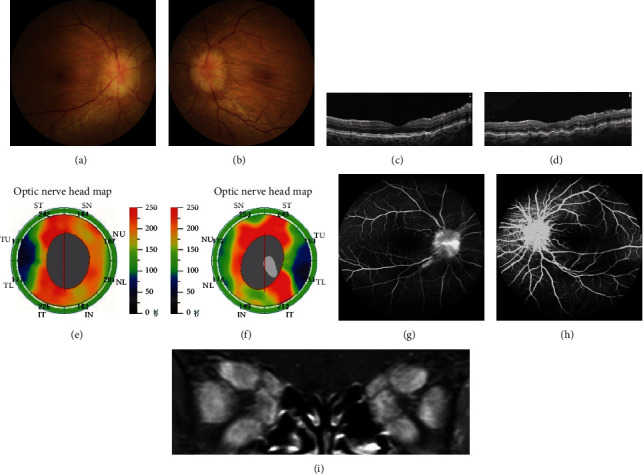
TAO patient a 50-year-old female who presented with bilateral CRFs and optic nerve edema. (a, b) The fundus photograph of bilateral CRFs with optic nerve edema. (c, d) The OCTA of bilateral CRFs in macula. (e, f) Bilateral CRF optic nerve edema of optic nerve head in the OCTA finding. (g, h) The bilateral CRF optic nerve edema of optic nerve head in the FFA finding. (i) MRI represents the crowded orbital apex and phlegmonosis from the enlarged extraocular muscles.

A total of 13 DON with CRF patients (24 eyes) were enrolled in this study, and they are listed in Table 1. Fifteen of the 24 eyes (62.5%) showed optic disc edema (ODE group), and representative images are shown in Figure 1. The mean age and thyrotrophin receptor antibody (TRAb) level at presentation were 45.73 ± 12.14 years of age and 16.31 ± 11.00 U/L, respectively. The durations of autoimmune thyroid diseases and TAO were 9.60 ± 3.60 and 7.80 ± 3.65 months, respectively.

**Figure 2:**
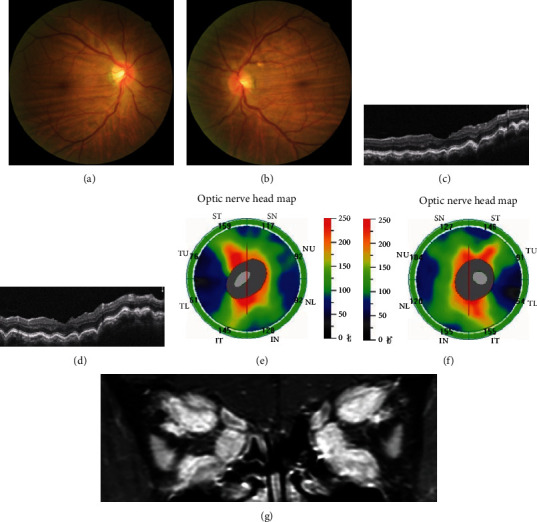
TAO patient of a 57-year-old male who presented with bilateral DON and CRFs. (a, b) The fundus photograph of bilateral CRFs. (c, d) The OCTA of bilateral CRFs in macula. (e, f) The OCTA of bilateral CRFs in optic nerve head; (g) MRI represents the crowded orbital apex and phlegmonosis from the enlarged extraocular muscles.

Nine of the 24 eyes (37.5%) without optic disc edema (NODE group) and representative images are shown in Figure 2. The mean age and TRAb at presentation in the NODE group were 48.00 ± 10.58 years of age and 10.41 ± 11.22 U/L, respectively. The durations of autoimmune thyroid diseases and TAO were 15.00 ± 9.25 and 11.22 ± 7.81 months, respectively.

Eight eyes (8/9 eyes, 89%) and 9 eyes (9/15, 60%) belonged to males who smoked in the ODE and NDE groups, respectively. One eye (1/9 eyes, 11% and 1/15 eyes, 7%) belonged to a diabetic patient in both groups. One eye (1/9 eyes, 11%), and 3 eyes (3/15 eyes, 20%) belonged to hypertensive patients in the ODE and NODE groups, respectively. The common primary thyroid disease of DON patients was hyperthyroidism. There was no difference in thyroid status at the time of diagnosis between the two groups.

### 3.2. Comparison of the Ophthalmic Examination Parameters at the Time of Diagnosis

The parameters of the ophthalmic examination of 13 DON with CRF patients (24 eyes) are listed in Table 2. The mean BCVA (0.06 ± 0.15 vs.0.29 ± 0.27) and VF-MD (−3.49 ± 1.56 vs.−6.55 ± 3.71 dB) were significantly different between the ODE and NODE groups (all *p* < 0.05). The RNFL (209.38 ± 110.09 *μ*m) in the ODE group was thicker than that in the NODE group (114.88 ± 21.02 *μ*m, *p* < 0.05). The GCCL (89.43 ± 7.37 *μ*m) in the NODE group was thinner than that in the ODE group (101.80 ± 8.39 *μ*m, *p* < 0.05). There was no difference in the parameters between either group, including the average IOP, proptosis, CAS, optic nerve length, number of enlarged EOMs per eye, or the axial length.

### 3.3. Comparison of the Ophthalmic Parameters after Orbital Decompression between the NODE and ODE Groups within 6 Months

**Figure 3:**
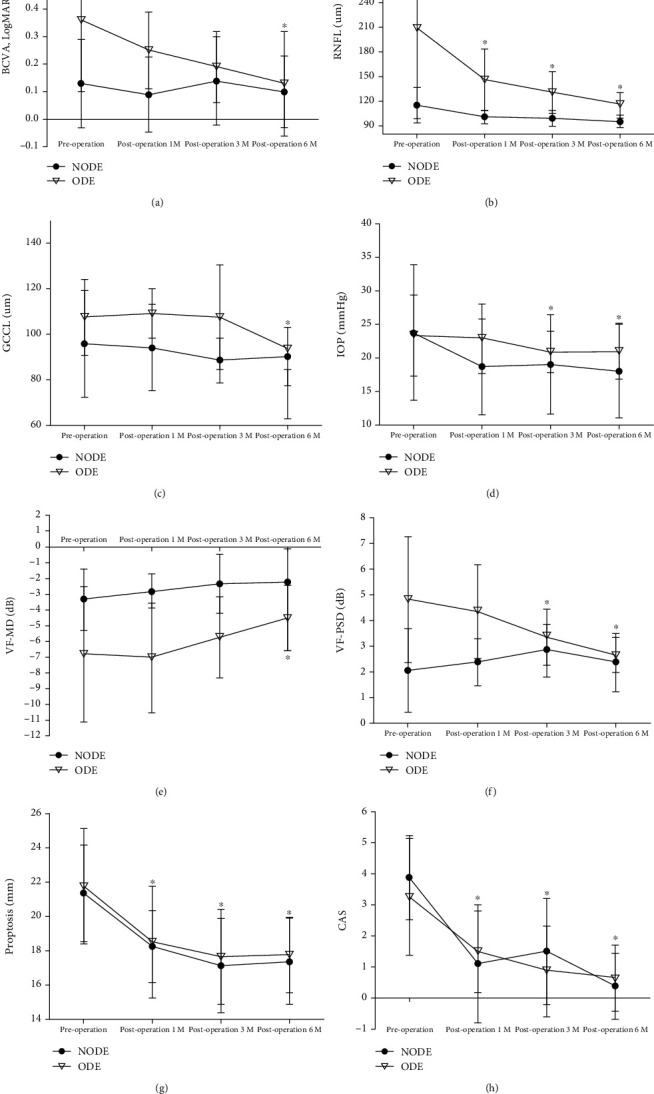
Changes of ophthalmic parameters after balanced medial plus lateral wall orbital decompression in the ODE (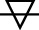) and NODE group (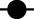) up to 6 months of follow-up. Continuous variables are presented as the mean ± SD. (a) The best corrected visual acuity (BCVA). (b) Retinal nerve fiber layer (RNFL). (c) Ganglionic cell layer (GCCL). (d) Intraocular pressure (IOP). (e) Mean deviation (MD) of the automated visual field. (f) Pattern standard deviation (PSD) of the automated visual field. (g) Proptosis. (h) Clinical activity scores (CAS). ^∗^Statistically significant.

Nineteen of 24 eyes experienced orbital decompress ion in our study, and valid data from 16 eyes within 6 months after the operation were collected (shown in Table 3 and Figure 3), including eight of 16 CRF eyes without ODE (NODE group) and the remaining eyes with ODE (ODE group). Among them, eight eyes (8/8 eyes, 100%) and five eyes (5/8, 62.5%) belonged to males in the NODE and ODE groups, respectively.

The generalized estimating equation (GEE) method was used for the two groups. There was a main effect in the ophthalmic parameters between the two groups, including RNFL (*p* = 0.001), GCCL (*p* = 0.039), VF-MD (*p* = 0.001), and VF-PSD (*p* = 0.04). The RNFL and GCCL in the ODE group were significantly thicker than those in the NODE group, and the VF-MD and VF-PSD in the ODE group were worse than those in the NODE group. There was no difference in any other parameters, including BVCA, IOP, proptosis, and CAS, between the two groups.

Compared to preoperation, RNFL, proptosis, and CAS were significantly decreased at each checkpoint (all *p* < 0.05). IOP (*p* = 0.039) and VF-PSD (*p* = 0.030) were significantly decreased at 3 months postoperation. Moreover, all parameters in both groups at 6 months postoperation were found to have significantly improved (all *p* < 0.05).

BCVA and RNFL at 6 months postoperation were found to have an interaction effect between the two groups. The improvement amplitudes of BCVA (*p* = 0.020) and RNFL (*p* = 0.039) in the ODE group were significantly greater than those in the NODE group. There was no difference in BCVA between the ODE group (0.13 ± 0.19) and the NODE group (0.10 ± 0.13); 4 eyes (4/8 eyes, 50%) in the ODE group and 2 eyes (2/8 eyes, 25%) in the NODE group experienced partial vision loss (BCVA, logMAR ≥ 0.2). The disc edema of all eyes (8/8 eyes, 100%) in the ODE group was completely mitigated after orbital decompression. The CRF resolution of 2 eyes (2/8 eyes, 25%) in the ODE group and no eyes (0/8 eyes, 0%) in the NODE group was mitigated.

## 4. Discussion

DON is the most frequent vision-threatening condition of TAO, an autoimmune disorder that predominantly affects patients with hyperthyroidism [16]. CRFs were first described in 1884 by Nettleship and have often been regarded as an indicator of visually threatening situations in TAO patients, such as DON [5]. Moreover, ODE, a specific indicator of DON, has been described in a EUGOGO survey and may be secondary to the inflammation and edema that coexist in the orbit [10, 11]. Therefore, visual impairment is hypothesized to be more severe than that of only CRFs if TAO patients have CRFs and ODE. Not surprisingly, the present study showed worse mean BCVA and VF-MD in the ODE group than in the NODE group. These outcomes confirmed that visual impairment in patients with CRFs with ODE is more severe than that in patients with CRFs only.

Our study showed that the peripapillary RNFL was thicker in the ODE group than in the NODE group. Similar results have been reported in patients with TAO at different stages of the disease compared with healthy subjects [11]. However, it was previously reported that DON causes progressive thinning of the RNFL, which is highly correlated with visual function [17]. Moreover, a previous study showed that the RNFL was significantly thinner in chronic DON eyes than in acute DON eyes [18]. The explanation for this significant difference is that the patients in these studies were primarily TAO patients at different stages of the disease.

Previous studies have reported that DON causes thinning of the GCCL, which is highly related to visual impairment [17, 18]. However, our study showed that the thickness of the GCCL in the ODE group was greater than that in the NODE group. One possible reason is that axon swelling of ganglion cells was caused by altered translaminar pressure gradients from increased orbital pressure in our study, despite GCCL atrophy in DON patients in previous studies.

CRFs with ODE have rarely been described in TAO patients in a few case reports [5, 6]. The mechanisms of CRFs with ODE are not clearly understood but are hypothesized to be related to orbital hypertension, which may place compressive stress on the choroid, Bruch's membrane, and optic nerve; the latter results may be related to visual dysfunction [6]. Our study showed that there was no difference in the following parameters of orbital hypertension between the ODE and NODE groups: elevated IOP, optic nerve length, enlarged EOM, axial length, and worse proptosis. These results highlighted that pressure-based etiology may play a role in the development of CRFs and ODE in our study.

Inflammation is another etiology hypothesized to be related to CRFs with ODE, which may be secondary to orbital inflammation and edema in TAO patients [11]. In this study, the results showed that 63% of DON eyes (15/24 eyes) had optic disc edema and 67% of them (10/15 eyes) had positive activity. These results highlight that inflammatory-based etiology plays a role in the development of CRFs with ODE. Moreover, optic disc swelling at diagnosis among DON patients was highly predictive of unresponsiveness to intravenous steroids, which was confirmed by a previous study [19]. In this study, the majority of DON patients were unresponsive or poorly responsive to intravenous steroids. Therefore, inflammation may be a complicating factor, and in these cases, urgent orbital decompression was performed in this study.

To our knowledge, orbital decompression for DON patients has been shown to be effective in alleviating CRFs and ODE and rescuing visual function. During a 6-month follow-up in our study, the results after orbital decompression showed that all parameters of the NODE and ODE groups were improved compared with the preoperative values at every time point, except for the mean BCVA at 3 months postoperation in the NODE group. Moreover, CAS and proptosis in both groups were significantly decreased at every time point compared with the preoperative values. Therefore, the coexistence of pressure- and inflammation-based etiologies may be the mechanism underlying CRFs with ODE. This coexisting etiology was reported in a previous study [11].

At 6 months postoperation, our study showed that all ODE eyes (8/8 eyes, 100%) and the CRFs of 2 eyes (2/16 eyes, 12.5%) in the both group were mitigated. Tran et al. showed CRF resolution after TAO treatment in 30% eyes (5/17 eyes) [5]. These studies indicated that CRFs could be effectively alleviated if treated properly. However, VF-MD in the ODE group was significantly worse than that in the NODE group, and 4 of 8 eyes (50%) in the ODE group experienced partial vision loss (BCVA, logMAR ≥ 0.2), more so than in the NODE group (2/8 eyes, 25%). Of note, DeMaria et al. showed that one patient with CRFs, a thicker RNFL and macular ganglion cell loss, complained of visual loss after orbital decompression [9]. These results indicated that CRFs with ODE may portend an increased risk of worsening vision compared with CRFs from orbital decompressive surgery. The possible explanation was that partial visual acuity or field loss was attributed to long-standing untreated compressive optic neuropathy and the stress of orbitotomy. However, Tran et al. showed that all five eyes with ODE recovered their vision after orbital decompression [5]. The possible reason was that the duration of optic disc compression was relatively short in these patients, who received timely and effective treatment.

Some limitations of this retrospective study were as follows: First, all of the patients were evaluated by one ophthalmologist, and the orbital decompressions were executed under the care of two of the authors, which could improve the reliability but might create systematic bias. Second, disease progression was not taken into account because different disease stages might influence the outcome. Third, due to the small sample size, a larger study is necessary to confirm the conclusion, although some of our results reached a level of significance.

## 5. Conclusion

Whether CRFs relieves or not, balanced orbital decompression for DON patients can significantly improve visual functions and eliminate optic disc edema.

## Figures and Tables

**Table 1:** Clinical characteristics of chorioretinal folds (CRFs) in DON patients.

Variables	CRFs *n* = 13 patients, 24 eyes		*p*
	NODE *n* = 9 eyes	ODE *n* = 15 eyes	
Age (years)	48.00 ± 10.58	45.73 ± 12.14	0.617
Eyes belonging to male/female	8/1	9/6	0.132
Eyes belonging to smokers (yes/no)	8/1	9/6	0.132
Eyes belonging to diabetic patients (yes/no)	1/8	1/14	0.677
Eyes belonging to hypertensive patients (yes/no)	1/8	3/12	0.509
Eyes belonging to thyroid status at diagnosis (hyperthyroidism/normal/hypothyroidism)	4/4/1	7/5/3	0.861
Eyes belonging to Graves' disease	9/9	15/15	NA
Treatment for thyroid diseases before visit us (antithyroid drug)	9/9	15/15	NA
TRAb (*μ*/L)	10.41 ± 11.22	16.31 ± 11.00	0.217
Graves' disease duration (months)	15.00 ± 9.25	9.60 ± 3.60	0.122
TAO duration (months)	11.22 ± 7.81	7.80 ± 3.65	0.292

TRAb: thyrotrophin receptor antibody; NA: not applicable; *p* ≤ 0.05 means statistically significant.

**Table 2:** Ophthalmic parameters of chorioretinal folds (CRFs) in DON patients at diagnosis.

Variables	CRFs *n* = 13 patients, 24 eyes		*p*
	NODE *n* = 9 eyes	ODE *n* = 15 eyes	
BCVA (logMAR)	0.06 ± 0.15	0.29 ± 0.27	**0.008**
VF-MD (dB)	−3.49 ± 1.56	−6.55 ± 3.71	**0.006**
VF-PSD (dB)	3.06 ± 1.96	4.42 ± 2.45	0.169
P100 latency (ms)	109.38 ± 9.49	110.64 ± 10.89	0.800
P100 amplitude (lV)	5.63 ± 2.39	4.98 ± 1.30	0.493
IOP (mmHg)	23.67 ± 9.57	20.27 ± 6.68	0.417
Proptosis (mm)	20.67 ± 2.29	21.20 ± 4.59	0.751
Axial length (mm)	23.79 ± 1.09	24.06 ± 1.25	0.489
Optic nerve length (mm)	38.17 ± 3.58	36.24 ± 3.67	0.243
Number of enlarged EOM per eye (eyes, %)	5.00 ± 0.71	4.73 ± 0.70	0.395
RNFL (*μ*m)	118.11 ± 17.26	191.07 ± 81.52	**0.002**
GCCL (*μ*m)	89.43 ± 7.37	101.80 ± 8.39	**<0.001**
CAS	2.44 ± 1.94	3.27 ± 1.71	0.323
CAS ≥ 3 (eyes, %)	4, 44%	10, 67%	0.356

*p* ≤ 0.05 means statistically significant.

**Table 3:** Comparison of ophthalmic parameters after orbital decompression in the chorioretinal fold (CRF) patients with optic disc edema (ODE group) and without ODE (NODE group) within 6 months.

Variables	Main effect				Interaction effect		
	*p*(group)	*p*(time 1)	*p*(time 2)	*p*(time 3)	*p*(group∗time 1)	*p*(group∗time 2)	*p*(group∗time 3)
BCVA (logMAR)	0.056	0.055	0.142	**0.013**	0.322	0.062	**0.020**
RNFL	**0.001**	**0.015**	**0.009**	**0.006**	0.093	0.054	**0.039**
GCCL	**0.039**	0.972	0.531	**0.004**	0.643	0.541	0.186
IOP	0.429	0.050	**0.039**	**0.017**	0.079	0.500	0.294
VF-MD	**0.001**	0.797	0.060	**0.022**	0.432	0.932	0.399
VF-PSD	**0.040**	0.070	**0.030**	**0.001**	0.747	0.088	0.064
Proptosis	0.738	**<0.001**	**<0.001**	**<0.001**	0.899	0.926	>0.999
CAS	0.777	**<0.001**	**<0.001**	**<0.001**	0.321	>0.999	0.301

Note: time 1, time 2, and time 3 represent the comparison of 1 month, 3 months, 6 months, and preoperation, respectively. *p* ≤ 0.05 means statistically significant.

## Data Availability

The datasets used and/or analyzed during the current study are available from the corresponding authors upon reasonable request.
